# What makes or mars the facility-based childbirth experience: thematic analysis of women’s childbirth experiences in western Kenya

**DOI:** 10.1186/s12978-017-0446-7

**Published:** 2017-12-29

**Authors:** Patience A. Afulani, Leah Kirumbi, Audrey Lyndon

**Affiliations:** 1 School of Medicine, University of California, San Francisco, USA; 2 Kenya Medical Research Institute, Nairobi, Kenya; 3 School of Nursing, University of California, San Francisco, USA

**Keywords:** Childbirth, Facility delivery, Experience, Person-centered care, Quality of care, Kenya, Sub-Saharan Africa

## Abstract

**Background:**

Sub-Saharan Africa accounts for approximately 66% of global maternal deaths. Poor person-centered maternity care, which emphasizes the quality of patient experience, contributes both directly and indirectly to these poor outcomes. Yet, few studies in low resource settings have examined what is important to women during childbirth from their perspective. The aim of this study is to examine women’s facility–based childbirth experiences in a rural county in Kenya, to identify aspects of care that contribute to a positive or negative birth experience.

**Methods:**

Data are from eight focus group discussions conducted in a rural county in western Kenya in October and November 2016, with 58 mothers aged 15 to 49 years who gave birth in the preceding nine weeks. We recorded and transcribed the discussions and used a thematic approach for data analysis.

**Results:**

The findings suggest four factors influence women’s perceptions of quality of care: responsiveness, supportive care, dignified care, and effective communication. Women had a positive experience when they were received well at the health facility, treated with kindness and respect, and given sufficient information about their care. The reverse led to a negative experience. These experiences were influenced by the behavior of both clinical and support staff and the facility environment.

**Conclusions:**

This study extends the literature on person-centered maternity care in low resource settings. To improve person-centered maternity care, interventions need to address the responsiveness of health facilities, ensure women receive supportive and dignified care, and promote effective patient-provider communication.

## Plain English summary

Maternal mortality remains a pressing problem in sub-Saharan Africa. Poor person-centered maternity care, which emphasizes the quality of patient experience, contributes both directly and indirectly to the poor outcomes. Yet, few studies in low resource settings have examined what is important to women’s childbirth experience from the women’s perspective. In this paper, we examine Kenyan women’s positive and negative facility–based childbirth experiences to identify aspects of care that are important to them. Our analysis shows that how women are received, cared for, and talked to in the facility, make a difference to their childbirth experience. Women had a positive experience when they were received well at the health facility, treated with kindness and respect, and given sufficient information about their care. The reverse led to a negative experience. These experiences were influenced by the behavior of both clinical and support staff and the facility environment. The findings of this study can help stimulate discussion between various stakeholders on how to promote positive childbirth experiences for women in Kenya, and elsewhere in sub-Saharan Africa.

## Background

Maternal mortality remains high in sub-Saharan Africa (SSA), despite improvements in the last decade [1]. The estimated maternal mortality rate in SSA in 2015 was 546 maternal deaths per 100,000 live births—accounting for approximately 66% of global maternal deaths [1]. Skilled attendance at birth is critical to reduce maternal mortality, as about three quarters of maternal deaths occur from complications around the period of birth [2]. Most efforts to improve maternal health in SSA therefore emphasized increasing the proportion of women delivering in health facilities with skilled attendants. However, maternal mortality has remained high in many countries despite increasing facility deliveries [3, 4]. This has highlighted the need to focus on the quality of facility-based delivery care [5]. Furthermore, documentation of disrespectful, abusive, and neglectful treatment of women during childbirth in facilities has highlighted gaps in person-centered maternity care [6–9].

Person-centered maternity care (PCMC) refers to maternity care that is respectful of and responsive to childbearing families preferences, needs, and values [10]. PCMC emphasizes the quality of the patient experience, and includes system and provider responsiveness, patient-provider communication, interpersonal treatment, and patient engagement [11, 12]. Poor PCMC can affect outcomes and deter women from seeking health services [9]. The experience of poor PCMC, even by a few women, leads to poor community perceptions of facility-based delivery care, which discourages many women from delivering in health facilities [13–15].

In Kenya, approximately 61% of women give birth in a health facility [16]. However, among women with no education and those in the lowest wealth quintile, only about 25% and 31% respectively give birth in a health facility, compared to 85% and 93% among women with secondary or higher education and those in the highest wealth quintile respectively [16]. Disparities in PCMC, in addition to differential access (physical and financial) and perceived need, may be driving these disparities in facility deliveries, as women of low socioeconomic status are more likely to be mistreated in health facilities [17].

Disrespect and abuse during childbirth are some of the most overt forms of poor PCMC, hence their prominence in the literature. However, to achieve true PCMC, a broader understanding of women’s experiences of care is important. This study extends the literature on PCMC by analyzing the range of women’s facility-based childbirth experiences to more holistically assess quality of care from their perspective. The aim of this study is to examine women’s facility–based childbirth experiences in a rural county in Kenya, to identify aspects of care that contribute to a positive or negative birth experience. The findings will guide the provision of care that is respectful of and responsive to women’s preferences, needs, and values.

## Methods

### Setting

This report stems from a larger study examining community perceptions of maternity care quality in a rural county in western Kenya. The County has a population of about one million and an estimated 40,000 annual births [18]. Approximately 43% of the population lives below the poverty line and only about 3% of women of reproductive age have more than a secondary education. About 24% of childbearing women are aged 15–19 years [16]. The County is divided into 8 sub-counties. One County Hospital—the only government facility that can conduct cesarean sections and with a functioning newborn unit—serves as a referral hospital to 7 sub-county hospitals, 18 health centers, and several dispensaries. There are also a number of faith-based and private health facilities in the county. The number of nurses, clinical officers, and doctors per 100,000 people in the county is 32, 19, and 4 respectively [19]. About 53% of births in the County occur in health facilities [16].

### Data collection

We conducted eight focus group discussions—one in each sub-county—with mothers 15 to 49 years old who gave birth in the 9 weeks preceding the study in 2016. We selected a focus group design based on our field experience in the region, which suggested women are more willing to discuss their experiences in groups with their peers than with an individual interviewer not familiar to them. Focused groups are believed to create a socially oriented environment and a sense of belonging to a group, which can increase participants’ sense of cohesiveness and help them to feel safe to share information [20, 21]. We chose the time frame of within 9 weeks postpartum based on the desire to capture women’s recollections in close proximity to their births, while also balancing feasibility and subject burden. Three to six focus groups are usually adequate to reach data saturation [20, 21]. To recruit respondents for each sub-county, we randomly selected one health unit that had not been selected for other aspects of the larger study. The health unit’s community health volunteers then recruited eligible women. Each woman was screened for eligibility, given information about the study, and asked for written informed consent. We provided snacks and gave each woman 200 KES (~$2) for transport.

Discussions involved six to ten women in each group and lasted about 90 min. Each group consisted of women from a single health unit and most (but not all) of the women within the group gave birth in the same facility. But the eight groups represented women who gave birth in different health facilities across the 8 sub-counties. Two-trained Kenyan female research staff moderated each group; one led the discussion using a discussion guide, and the other took notes and managed audio recording. The moderators were trained for this study and were not affiliated with the facilities. One moderator was a trained midwife who was no longer practicing; the other had a social science bachelor’s degree. The open-ended discussion guide asked women to tell their birth stories, describe their best and worst facility-based delivery experiences, their expectations for care, and what made them feel welcomed in a facility. Discussions were conducted in Swahili or Luo. The first discussion was held in a private space in a health facility, with the remaining seven discussions held in private spaces in the community. The discussions were moved to the community after the first group because the moderators noticed women were not comfortable discussing their experiences in the health facility. The community health volunteer helped to identify a private space in the health unit that was accessible to all the women invited to participate in the focus discussion. All women in a given focus group came from one health unit so did not have to travel long distances for the interview. Discussions were audio recorded, and simultaneously translated and transcribed by the research staff. Ethical approval was obtained from the University of California, San Francisco Committee for Human Subjects Research and the Kenya Medical Research Institute Scientific and Ethics Review Unit.

### Data analysis

We analyzed the data thematically following the approach of Braun & Clarke (2006) to identify, analyze, and report patterns within data. We took a constructionist approach—meaning we assumed women’s descriptions of their experiences are socially produced [22]. We coded data inductively, considering both the semantic (surface) and latent (underlying) meaning of the text, and focusing on salience rather than frequency. We iteratively read and re-read the transcripts and coded line-by-line across the entire data set. Two coders (the first author and a study assistant) double coded half of the transcripts and compared codes to check consistency. We then analyzed initial codes to generate categories and identify themes [22]. We compared our themes to the experience of care dimensions of the WHO quality of care framework for maternal and newborn health to guide their naming. The framework highlights three domains—effective communication, respect and dignity, and emotional support—under experience of care, which influence people-centered outcomes [23]. Throughout the process, we wrote analytic and reflexive memos to capture emerging ideas and examine our assumptions, preconceptions, and reactions to the data. We used Atlas.ti to support data management and analysis.

## Results

Fifty-eight women participated in the eight focus groups. The demographic characteristics of the study participants are shown in Table 1. We identified four factors influencing women’s perceptions of the care they received: responsiveness, supportive care, dignified care, and effective communication.

**Table 1:** Characteristics of respondents (*N* = 58)

Variable	*N*	%
Age: mean (SD)	58	26(6.6)
Parity: mean (SD)	58	4(2.0)
Marital status		
Single	6	10.3
Married	51	87.9
Widowed	1	1.7
Delivery place		
Government Hospital	27	46.6
Government Health Center	23	39.7
Mission/Private Facility	5	8.5
Other	3	5.2
Previous facility delivery	47	90.4
Education		
None	1	1.7
Primary	45	77.6
Secondary/Vocational	10	17.2
College	2	3.4
Occupation		
Agricultural labor	23	39.7
Salaried worker	4	6.9
Self-employed	5	8.6
Other	4	6.9
Unemployed	22	37.9
Religion		
Catholic	12	20.7
Protestant/Pentecostal	22	37.9
Seventh Day Adventist	14	24.1
Other Christian	7	12.1
Other religion	3	5.2
Tribe		
Luo (County’s dominant tribe)	38	65.5
Kuria	12	20.7
Other	8	13.7

### Responsiveness: From care denial to a warm reception

We use responsiveness to describe how women are received when they arrive at the health facility. The responsiveness of providers and the whole system made a difference to women’s experiences and perceptions of quality of care. Many women’s descriptions of their best experiences related to how they were received when they arrived at the facility, starting from the facility gate. Women often travelled long distances to reach facilities. Being welcomed in a friendly and caring manner and seeing that providers appreciated they needed immediate care and were hastening to help them upon arrival contributed to a positive experience. Women appreciated providers going out of their way to make sure they were received well, even if this pointed to other system failures, such as providers keeping personal drugs and supplies because they are not always readily accessible from the facility.

*“[The best thing was] the good welcome…. I arrived in the hospital at 1.00 am at night, the watchman opened the gate well, he welcomed me and called the nurse, then she welcomed me also…when I arrived I found everything ready. She took me to the bed, did everything, told me now it is late the chemist is closed, we do not have any drug here but she looked for some of her personal drugs and used them on me... She took her time to be with me and handled me well. This made me to say it is good to go to the hospital to deliver.”*

The watchman (security man) played an important role in a facility’s responsiveness, and the extent to which the watchman was responsive was pivotal to women’s positive and negative care experiences. The watchman was often the first person the woman came into contact with at the health facility, which was often locked at night. His manner at the gate was therefore core to how the women felt she was received. In one case, the unresponsiveness of the facility, including a locked door without someone to man it, the watchman either not available or intentionally refusing to open the gate, and a provider not being readily available, led to a woman giving birth outside the facility gates, which she described as her worst experience.

*“[w]hen my time for delivery reached, it was at around eleven in the night. So when I came…I tried to knock the gate and even shout at the watchman to come and open for me but he did not. So I gave birth right at the gate… the doctor came at half past midnight and… the baby’s umbilical cord was cut outside at the hospital gate….”*

Women wished to be examined promptly upon arrival to the facility, and were unhappy when they arrived at transition periods, and had to wait till the next person on duty came to attend to them.

*“[W]hen I arrived at the health facility around 5pm in the evening, I just sat there and no one was ready to attend to me, they made a comment that if I want to deliver the baby I can just do it where I am sitting. So I [waited for the night nurses as] the ones on daytime duty do not want to attend to me. When I saw the night nurse reporting I went to talk to her, she then told me to come inside, she examined me and from there it did not take even two hours, then I gave birth. After that I felt good.”*

There seemed to be a mismatch between recommendations for women to seek timely care and how they were received when they arrived. Women reported providers telling them they were not far advanced in labor or accusing them of exaggerating their pain when they arrived early in labor. This discouraged them from seeking timely facility-based care.

*“Some of these hospitals when you go they keep on telling you that your time is not ready and you are just exaggerating. [It] makes some women to be discouraged and say that it is better [to] just go to a traditional birth attendant...”*

The most extreme form of non-responsiveness was when women were refused care and turned away from the health facility—even when they expressed fears that birth was eminent. Women often feared that they or their babies might die while trying to reach the next facility.

*“After walking everywhere [to several facilities], one nurse [at facility name] told me just to deliver even on the way. …[She] told me they are on strike. She was misbehaving and telling me if I want to deliver I can even deliver at the door, then she closed the door on my face... I pleaded with her to help me because I was feeling the baby near; it forced the motorbike person to take me back to look for [another] place I could deliver. As we were looking for places, I was just closing my legs [to hold baby in]…because I was feeling the baby near. I almost killed the baby.”*

Reasons why women were being turned away from health facilities included the following. First, around the time of this study there had been intermittent strikes by doctors and nurses in public health facilities in Kenya, with public facilities not providing services (our data collection was completed prior to the major strike by doctors and subsequently by nurses in Kenya which lasted several months [24]). Women who went to the public health facilities during the intermittent provider strikes were therefore likely to be turned away. Second, it seemed some facilities offering care for birth did not provide 24 h services, such that providers refused to admit women presenting towards the end of the day. Third, providers sometimes used poor infrastructure as a reason for denying care, as some women said they were turned away because the providers said they had no electricity. However, in some cases the reason was not apparent to women.

*“I went to [X facility] and said I will never go back [there], because when I went, they refused to open the door for me and on the other side I see them opening the door for another lady. So I just sat there with labor pains and pleading with them to open the door or call for me the nurse who is opening the door for another lady but they refused. The motorbike person now told me to go up somewhere and look for a place to deliver. We went to that place up there and it was a Pharmacy (chemist), we found it closed and then again we tried to come back to [X facility] but I said we go to [a private facility …. [at the private facility] I pleaded with them and they agreed to help me. They did examination and helped me just that moment.”*

Care refusal signaled to women that providers did not care about them. Motorbikes were the most frequent form of transport for women in labor, which made being asked to go to another facility when one was close to giving birth particularly dangerous and uncomfortable. One woman actually gave birth on the road after being turned away from the first facility she went to.

*“I went to [facility1] for delivery, they were not bothered with my presence…. I told them that the baby was near I might not reach [facility2]. They told me their time is up I should go to [facility2]. When I reached the way I was put on a motorbike. The motorbike person said he cannot carry me because blood had started coming out... He went and abandoned me somewhere on the way. I just sat on the way struggling alone until somebody was passing and I asked him for a phone, I called my husband, he came and as we reached the tarmac, I delivered on the way….they turned me away instead of helping me and if I died I could have said that these nurses do not care about people’s lives.”*

### Supportive care: From neglect and abandonment to caring and compassionate care

Supportive care includes aspects related to provider behavior and the health facility environment that affect women’s experiences after they have been admitted to the facility. The continuum of provider behaviors contributing to women’s experiences ranged from fundamental things providers did that made women feel supported to overt neglect. Some women’s descriptions of their best experiences suggested providers were caring, kind, compassionate, and helpful. Women appreciated it when they received routine clinical care such as: managing pain and bleeding, weighing the baby and conveying the baby’s weight, giving the baby immunizations, etc.

*“...So the best I experienced was they gave me injection to control too much bleeding and when I was leaving I was given some drugs. I was also given [a] birth notification…to help me get the [baby’s] birth certificate.”*

Lack of pain management on the other hand tended to contribute to a negative experience, especially when women felt providers did not care or were disrespectful.

*“Sometimes [they don’t give you any medicine when] you are in pain and the nurse is just looking at you like this. Sometimes we women do not respect each other… the nurse will say that the time you were looking for this baby with your husband I was not there so I will only come once you are ready to deliver, but they should be helping you and that is why you go to them for assistance.”*

Women appreciated it when providers were attentive to their basic needs like toileting, helping them get into bed after birth, offering tea for sustenance, and helping them breastfeed.

“*[T]he nurse who was there in the morning … she could wake us the mothers to go and bathe, later she made sure that after bathing you change to clean clothes, she treated us so well like the baby is hers… she ensures that before breastfeeding the baby your hand [is] clean.”*

Not having something to eat or drink after birth negatively affected the birth experience, as women reported often not eating for as long as two days while in labor. Lack of sustenance was a major concern in one focus group involving several women who delivered in the same facility.

*“…. they were taking tea…. and I was looking at them and no one was giving me… ….you really feel like drinking tea with them because you are too hungry that you can even fall…but no one bothers. (All chorus) yes…here [at this facility] nothing is given…”*

Women felt neglected when providers did not check frequently on them, did not respond promptly to their calls for help, or referred them to another facility after a period on admission without offering much help.

*“…instead of being with the patient they [nurses] were just on the other side while patients were suffering. It went like that [for 13 hours]. I was just the same during this time and [then] it was like the baby was almost coming out but could not and I was also wailing…. They now called [referral facility] people to come and take me there…when I arrived there the nurse I found was good, she was not bad…. [she] assisted me to deliver the baby as I arrived at 10.00 pm...I swore never to go back to [first facility]”*

Supportive care includes having a support person of one’s choice present during labor and birth. Although not all women wanted support persons with them, generally those who had a support person were happy to have this person there to help meet their needs when providers were unavailable. Those who were denied someone of their choice were dissatisfied.

*“They allowed the relatives to be with us ….I felt good because there are times you can be going to bath, and so they will remain and take care of the baby for you.”*

Being discharged too quickly was another source of concern to some, although other participants were happy they did not stay at the facility for long. Reasons for early discharge included insufficient beds or facility not providing 24 h services.

*“The worst I experienced in the hospital, I was discharged too quickly before I rested, because time was up…They [providers] were closing the health center as they were leaving and there is no ward there. Though I experienced the best there at first, later I was discharged too soon before resting.”*

Some women’s experiences were also negatively affected by lack of caring by non-clinical staff such as the people serving food—including refusal of food if the women did not have the utensils to be served with or if they were unable to go and serve themselves.

*“[If] you are not able to go pick your food …they keep on shouting from where they are standing that food, food, food...and you wonder how you will reach there because you are unwell and maybe in pain.”*

### Health facility environment and supplies

The health facility environment was deeply intertwined with women’s experiences. The environment affected receipt of supportive care indirectly through providers’ behavior (e.g., when providers used lack of resources as a reason for care refusal); and directly through structural resources such as cleanliness, availability of water, and adequate beds. Women’s worst experiences included being put on beds that were uncomfortable, had stained sheets, or were soiled with blood; having to share beds with other women; and going home after childbirth without bathing because of lack of water. A clean bed and water (particularly warm water) to bathe after they gave birth thus made a big difference.

*“It was a place well taken care of…the toilets were clean, where the baby was put was clean, and even where I was sleeping…here, you were given clean water and warm water for bathing [unlike other facilities] and so I liked the hospital a lot.”*

When women were asked what they would change in the health facility to make their experiences better, having more beds came up strongly.

*“If I am in [facility name], I will make sure there are more beds where mothers are sleeping, so that three or four mothers with babies do not share the bed. Imagine a mother who has just delivered, all the pain, also bleeding, how can you stand with that blood the whole night oozing because you cannot [sleep]. The mother is supposed to sleep next to the baby for the baby to feel the mother’s warmth to help baby learn to breastfeed. This is not possible when you are standing because the ward and beds are full.”*

The perception of being safe and comfortable is important to the birth experience. Women expected the facilities to have mosquito nets (malaria is endemic in the region), yet nets were not always available and some reported mosquitos bit their babies during their stay in hospital. In an extreme case, one woman described a terrible experience of being taken to a post-delivery room with uncovered windows and no light, which greatly affected her facility birth experience.

*“[A]fter delivery there is a room we were taken to sleep, there was no light, no windows, no beddings and we were to stay there feeling cold till morning. That is the worst I experienced… [The room had windows with no glass in them], and it was very cold and we were about three mothers with newly born babies. Cats were just entering through that window and just walking in that hospital…there was lack of security.”*

Women also felt facilities should supply basic items needed for their care rather than expecting them to buy medications, cleaning agents, sanitary supplies, etc. when they arrived in labor.

*“If I am given the opportunity…I will try hard so that things like Omo, cotton wool should be provided by the hospital not patients going to buy. The hospital should also have enough drugs instead of sending somebody to the Chemist to buy drugs while you have paid the facility.”*

### Dignified care: From abusive care to respectful care

Every woman deserves the right to dignified and respectful care. What women experienced as dignified and respectful care overlapped with what we considered supportive care. For example, some women felt respected when providers were attentive to their needs.

*“They treated me with respect, because they took good care of me until I delivered and did everything good. After delivery they gave me water for bathing, later I was taken to the bed and they gave me the baby to breastfeed.”*

The manifestations of undignified care were more explicit. For example, women’s experiences were negatively affected by lack of privacy and unnecessary physical exposure during examinations.

*“In the hospital … they are making you lie on the bed to be examined, there is no sheet to cover you and … other people are passing there. They will uncover you and sometimes you remain with a petty coat or sometimes you do not have one, you remain with a panty and they have not covered you, this sometimes embarrasses me...”*

Verbal abuse from providers also negatively affected the birth experience.

*“You are not given [a sanitary pad so] you bleed on the floor. When sister [nurse] comes she will start shouting at you ‘What is this? So you have brought your filthy nature from home to this hospital?’ …it may make you not to want to go back to such a facility.”*

Very few women reported being personally physically abused, but they saw others being physically abused, and this negatively affected their experience.

*“While she was laboring, the nurse shouted at her and even slapped her telling her that ‘I was not there while you were getting this baby and know that I can discharge you so that you go and deliver at home’ and I did not like the way she talked to this lady.”*

While the focus group discussion was directed to the birth period, women referenced abusive care during antenatal care. They complained of providers speaking harshly if they arrived late for antenatal care, and sometimes asking them to go home and come back another day—without concern for how far they had to travel to get to the facility. Such mistreatment influenced their decisions about where to give birth.

*“[I walked a far distance to the clinic with false labor] the nurse I found took my card and threw it away while quarrelling and asking me what time it was, whether it was the right time to come to the clinic. But I arrived at 11.00 am. She threw my card and this made me to think that if I deliver in this health facility I will not be treated well, so I decided to go and stay with my relative close to [another] Health facility.”*

Some women even reported what might be considered physical abuse during antenatal care.

“*The time of delivery I did not experience anything bad, but during the time of pregnancy when I was attending the clinic… there was a sister (nurse) who pinched me with the pen that I went to the clinic at a wrong time… Inside there was only one bench [which] was full, … I was also pregnant, standing and tired, then she pinched me with a pen saying why am I standing there. That did not make me feel good.”*

Another manifestation of non-dignified care is discrimination. Women had a positive experience when they felt they were not being discriminated against.

*“The person who assisted me was different, he had a lot of respect, did not care whether you are elderly or young, he treated me well.”*

But some women felt providers treated them or other women differently because of various attributes including their tribe, age, education, and wealth. In one case, a woman described a very non-responsive and non-supportive experience, with verbal abuse directed at her tribe, which negatively affected her birth experience.

*“[W]hen I arrived at the health facility, the nurse refused to examine me. …, I do not know whether she had been offended earlier by something else because she made a comment that she cannot examine me because Luos [a tribe in the county] are very stupid people. After her mentioning that I felt I should go away from this health facility but I just decided to stay until my husband came …. After this she told me to go back home then later we decided to do that. When I reached home labor pains started again so I decided to go back to the health facility and this time I found [a different nurse there]. When this other nurse was called to come and examine me, she was asleep, she continued sleeping and I also stayed up to morning…[I gave birth] without them touching me and [they] did not take care of me.”*

Women are acutely sensitive to providers’ “manners” and use of power. Providers’ behaviors either enabled women to open up and discuss issues bothering them or caused them to withhold valuable information about their condition. When providers were disrespectful, women reported withholding information from them.

*“You know that people have different attitudes… When you go to the clinic you study the nurses, and so when you come…and say that today I was unlucky because I found nurse so and so...[because] they are not treating the patients well,… When I come here if he asks me something [and] he has already talked to me rudely, then I will not talk.”*

On the other hand, when providers were respectful, kind, caring, polite, humble, and receptive, it put women at ease to be able to disclose and discuss concerns, and promoted a more trusting relationship. Such interactions influenced their future health-seeking behavior.

*“… when they talk to you in humility [and] take good care of you, this encourages you so that even if you become pregnant again you will not go to the TBA [Traditional Birth Attendant]... If you are treated well so that wherever you hear pain you tell them, even if you have a headache you will tell them. The way they treated us [positively] will force us to go back to them if we get another pregnancy.”*

### Communication: From no information to effective communication

How providers talked to women and what providers said (or did not say) directly and indirectly influenced women’s experiences. Responsiveness, supportive care, and dignified care were all influenced by communication. Some respondents mentioned “how” providers spoke to them made them feel welcome and reflected whether or not the provider was willing to care for them. Verbal abuse (described under dignified care) is one manifestation of how providers spoke to women.

An introduction sets the stage for the encounter, and knowing the name of the person caring for them was important. But most women said providers never introduced themselves—including some of the providers who they said were very good to them. In addition, while some did not mind being referred to in more generic terms like “mama,” most women preferred being called by their names, which they felt was more personal and respectful. They particularly did not like being referred to as “you.”

*“I think it is important for them to call you by your name because sometimes people are many in the facility and so when he calls you ‘wewe’[meaning ‘you’] then you will not be able to know whether it you who is being called or whether it is some other woman”*

Communication also includes the type, depth, clarity, and consistency of information provided. Poor communication due to unclear or too little information marred women’s experiences. Women were unhappy when they presented in labor but were asked to go home and return later because they were not advanced enough in labor. Inconsistent information and practices around whether or not women could stay at the facility when they presented early in labor also marred the birth experience.

*“When labor started I went to the health facility… but felt bad when they told me to go back home because my time for delivery is not yet. Another person came and asked me now you want to go back home and in the health facility, people are allowed to stay even for three days before they deliver…stay. After hearing this I went back and stayed. After that the nurse who told me to go home came and said, I told you to go home, go home. If labor increases and [you] have means you can even come back at night…. When I reached the gate the watchman returned me.…. When evening came the doctor on night duty asked me who told me to go home, when someone is in labor she is allowed to stay in the health facility for even three days…Please come so that I examine you. When he did examination on me I just delivered the baby there, so from there I felt good not like before.”*

Poor communication started from the antenatal period, as it reflected what women might have (or not have) been told during antenatal care. One gap in communication was whether or not women understood during antenatal care how to determine when they are in active labor and when to come to the health facility. In addition, women expressed frustration on being asked to provide sanitary items and detergents when they presented in labor. This frustration was because they had not been told during antenatal care to prepare to bring these items to the facility at the time of birth. They also sometimes did not understand why the items were being asked for in the stated quantities.

*“It could have been better if they tell us [at antenatal clinic] to buy Jik, Omo, Cotton Wool, so when you are carrying your card the time you feel labor pains you put them also in your bag and go with them. You know sometimes delivery time may come at a time when you do not even have any money so when they again put pressure that you buy these things [when you are in labor], this does not make us feel good.”*

Some women also felt they were not given enough information about their care, such as why they needed a cesarean section or referral, which made them feel not enough was being done for them.

“*They did not tell me anything, I just saw the vehicle ready and they told me you are going to [X referral hospital] because you are going for an operation.”*

On the other hand, women appreciated information about their care, including when providers told them findings of vaginal examinations and labor progress. Open communication and encouragement from providers led to a positive experience.

“*They were also next to me and told me that I will give birth at six in the morning and they were awake the whole night and asked me to call them anytime …they made me happy and this even reduced the amount of pain that I was going through because they kept on encouraging me and this made me feel welcomed”*

In addition, women appreciated information on how to care for themselves and their babies, including information on breastfeeding, nutrition, and when to resume heavy work after childbirth. Effective communication includes listening to women and giving them opportunities to ask questions. Not listening to women’ concerns impaired their autonomy and involvement in their care, which affected their birth experience. One respondent attributed her problems after birth to the provider not listening to her concerns about her preferred birth position.

*“I have never given birth while lying on my back so when I tried and while they were telling me to push, I was losing my breath. But when I tried to tell him how I am used to giving birth he refused to listen to me and that made me to take so long before giving birth and later I developed serious problem in my body.”*

Providers’ behaviors sometimes made it difficult for women to ask questions about their care. While some women felt confident to ask questions, many said they were afraid and were unable to speak up for themselves, even when they needed something.

“*I was so hungry and whoever escorted me had already gone back home so I wanted to ask her that, when someone gives birth you do not even give something to eat? But I was not able to...You are scared of asking because they will tell you that don’t you have your husband or your mother”*

## Discussion

This study explored women’s positive and negative childbirth experiences to identify factors that affect their perceptions of person-centered maternity care. We found issues key to women’s experiences included, how women were received at the health facility, how providers spoke to them and what was said, whether providers were kind and respectful to them, and the environment in which care was provided. The four main themes of our analysis were responsiveness, supportive care, dignified care, and effective communication. These themes are not mutually exclusive; they are overlapping domains of person-centered care used to provide an organizing framework for the data. The themes overlap with the experience dimensions of quality (effective communication, respect and dignity, and emotional support) in the WHO quality of care framework for maternal and newborn health [23]. Our study thus illustrates what the experience of care dimensions in the WHO framework might mean to women delivering in health facilities in Kenya. In addition, we present responsiveness as a separate domain to highlight the issues related to how women are received when they first arrive to a facility in a resource-limited setting.

PCMC is an important pathway to improve outcomes for mothers and babies [9, 12]. Most of the work in this area in SSA has focused on mistreatment [7]. We too find evidence of mistreatment in women’s descriptions of their worst experiences, with the most negative forms of poor PCMC or mistreatment in this study being care refusal, abandonment, and verbal and physical abuse. These have been documented in other studies in Kenya [25, 26]. Several factors account for such poor interactions between women and providers. These factors include inadequate training on person-centered care, an overburdened and underequipped health care system where providers are working under very stressful conditions, individual provider attitudes and their responses to the stressful work environment, patient-provider power and gender dynamics within a hierarchical society, and social norms regarding the acceptability of various behaviors [26–31]. Under these conditions, women’s experiences are often not a key concern of providers.

Our analysis extends the literature beyond mistreatment to also highlight factors that lead to a positive facility-based childbirth experience. Recognizing the aspects of care that women found helpful and respectful, as well as the aspects of care that they found negative, can help to tailor interventions to reinforce or change those aspects of care. For example, we find that the aspects of care that led to a positive experience for women were quite fundamental: receiving them in a timely and welcoming manner, providers introducing themselves to them, referring to them by names they prefer to be called, explaining examinations and findings to them, helping them understand their labor progress, putting them at ease to ask questions, listening to them, allowing support persons if they want one, a drink after childbirth, warm water to bath, their own bed with clean sheets and a mosquito net, providing routine care like pain medications, etc. These aspects of care that positively affected women’s experiences provide specific targets for quality improvement efforts that could be implemented at the facility level. In addition, it is easier to engage providers with these positive targets than from a perspective of mistreatment, where providers feel blamed and are more likely to be defensive. For example, providers cited forgetfulness (in our other work) as a reason why they do not do certain things such as introducing themselves to women. This expression of forgetfulness as a reason for poor PCMC is an easier entry point to affect change than deep seated power dynamics that are undoubtedly also at play.

Furthermore, women described variable experiences with different providers in the same facility or in different facilities. They often described a negative experience that became positive because someone else stepped in and the situation improved, or things got better when they went to a different facility. This inconsistent care, both within facilities and between facilities, highlights opportunities for improvement and further enquiry. That some providers working in the same conditions are able to provide care that leads to positive birth experiences (while care by others leads to negative experiences) suggests interventions to improve women’s experiences are possible within the existing infrastructure. Studies looking into why different providers behave differently under the same working conditions might help develop targeted interventions to improve women’s experiences.

Even though we present data under different themes, it is apparent from the supporting quotes that women’s experiences were hardly a result of one factor. Non-supportive care often started with poor responsiveness, and was associated with non-dignified care and poor communication. This implies interventions to improve women’s experiences will require addressing multiple domains of person-centered care. Given the role of non-clinical and support staff such as the watchmen, cleaners, and food servers in women’s experiences, quality improvement activities need include both clinical and non-clinical staff. Training of both clinical and non-clinical staff should emphasize how to interact with women and their families so they feel welcomed, supported, and respected.

Despite efforts promote facility-based childbirth, facilities exhibit varying levels of responsiveness to women’s needs, with some having very poor levels of responsiveness where women are ignored on arrival or even not granted access to the facility. Quality improvement efforts need to target effective triaging to ensure all women are fully examined on arrival and adequately monitored during labor. Where active labor has not started, providers need to discuss this with women and listen to their unique situations, especially considering the distances that women may need to travel to the facility, before making a decision as to whether they should be asked to go home and return when they are more advanced in labor. Policies related maternity units not providing 24 h services need to be revisited to assess their utility, to prevent women from being turned away because a facility is closing for the day. The effect of health care provider strikes is beyond the scope of this paper, although it is obvious how that affects women’s birth experiences and other health outcomes.

Another gap in women’s experience is due to poor patient-provider communication, starting from the antenatal period. Women’s dissatisfaction about being asked to go home and return when they were far advanced in labor raises questions about the effectiveness of antenatal communications regarding when to present for care and what to expect. Health literacy may be a contributing factor, and clinicians need to consider the gap between their and their patients’ understanding of health information. Women need to be given clear, consistent, and literacy-appropriate information about their health care to enable them to prepare adequately for birth. Most importantly, listening to women’s individual circumstances will help guide decisions on their care and help them to accept specific plans for their care. Improving patient-provider communication should be a priority in quality improvement.

Very few studies have rigorously evaluated interventions to improve PCMC in SSA [32, 33], hence the need for more studies in this area. Some of the approaches used have included provider training to increase provider knowledge of patient and provider rights, values clarification exercises with providers, empowering patients to demand better care, and setting up mechanisms to hold provider’s accountable for their actions [32, 33]. Facility managers could also put systems in place for women to have the opportunity to give feedback on their experiences (e.g. through an anonymous call system) and to acknowledge providers who are doing well through an award system. This might help motivate providers to treat women well. Interventions to address bias and promote positive stress coping behaviors among providers, as well as broader system level interventions to improve working conditions, may also improve women’s experiences. Low investment in the health care sector contributes significantly to poor infrastructure and a sub-optimal environment for care. Investments to improve the health facility environment and provision of basic supplies will thus help improve women’s experiences, as well as that of providers. Future studies into the types of interventions that will be feasible, acceptable, and effective in improving person-centered maternity care in low resource settings are needed.

## Limitations

A limitation of this study is that the qualitative nature of our study limits transferability: the findings are specific to our participants’ experiences and may not reflect the experiences of women in other settings in Kenya or even all women in the study County. In addition, women described their negative experiences in more detail than their positive experiences, although there were more instances of good than bad experiences. Thus, we note that some of the negative experiences may not represent the majority of the participants. However, they do reflect the experiences of some women, thus deserve attention. Finally, the study was designed to elicit information on women’s experiences and so does not represent their perceptions on the technical quality of care they received (however women referenced technical aspects of care that were obvious to them such as control of bleeding and pain).

Common ways of assessing trustworthiness include triangulation and member checking. These data are consistent with other data from our larger study, as well as with other studies on mistreatment during childbirth in Kenya [25, 26]. We did not have the opportunity to do member checking with the focus group respondents, but the findings were presented to relevant stakeholders including county officials, health workers, and community health extension workers from all the study sub-counties. The research staff were also present at these presentations. The results were received well by providers as reflecting issues in their facilities (although the extent might not have been apparent to them) and by the community health extension workers as some of the concerns women have raised with them. Similarly, the research staff, including some of those who were involved in the quantitative data collection, mentioned the qualitative data reflected stories women had shared with them.

## Conclusions

Our findings suggest that the things that make a difference to women’s childbirth experiences may rely on seemingly straightforward changes. Some of these changes (e.g. respectful communication) depend upon changes in provider attitudes. The findings of this study can help stimulate discussion between various stakeholders on how to promote positive childbirth experiences for women in Kenya, and elsewhere in SSA. Discussing findings with providers could serve as a starting point for implementing behavioral changes to promote PCMC. Other changes such as those relating to the environment and supplies require more systemic changes, which are possible with the commitment of health system leaders. It takes a whole system—beginning with the antenatal provider, through the watchman at the facility gate, to the person serving food on the ward—to make or mar the facility-based childbirth experience. PCMC quality improvement efforts should therefore involve all providers, including support staff, in a health facility.

## Abbreviations

PCMC: Person-centered maternity care
SSA: Sub-Saharan Africa
WHO: World Health Organization
